# Identification of a novel family of carbohydrate-binding modules with broad ligand specificity

**DOI:** 10.1038/srep19392

**Published:** 2016-01-14

**Authors:** Cheng-Jie Duan, Yu-Liang Feng, Qi-Long Cao, Ming-Yue Huang, Jia-Xun Feng

**Affiliations:** 1 State Key Laboratory for Conservation and Utilization of Subtropical Agro-bioresources, The Key Laboratory of Ministry of Education for Microbial and Plant Genetic Engineering, and College of Life Science and Technology, Guangxi University, 100 Daxue Road, Nanning, Guangxi, 530004, China

## Abstract

Most enzymes that act on carbohydrates include non-catalytic carbohydrate-binding modules (CBMs) that recognize and target carbohydrates. CBMs bring their appended catalytic modules into close proximity with the target substrate and increase the hydrolytic rate of enzymes acting on insoluble substrates. We previously identified a novel CBM (CBM_C5614-1_) at the C-terminus of endoglucanase C5614-1 from an uncultured microorganism present in buffalo rumen. In the present study, that the functional region of CBM_C5614-1_ involved in ligand binding was localized to 134 amino acids. Two representative homologs of CBM_C5614-1_, sharing the same ligand binding profile, targeted a range of β-linked polysaccharides that adopt very different conformations. Targeted substrates included soluble and insoluble cellulose, β-1,3/1,4-mixed linked glucans, xylan, and mannan. Mutagenesis revealed that three conserved aromatic residues (Trp-380, Tyr-411, and Trp-423) play an important role in ligand recognition and targeting. These results suggest that CBM_C5614-1_ and its homologs form a novel CBM family (CBM72) with a broad ligand-binding specificity. CBM72 members can provide new insight into CBM-ligand interactions and may have potential in protein engineering and biocatalysis.

Most carbohydrate-active enzymes are modular proteins that comprise two or more discrete catalytic modules (CMs) and non-catalytic carbohydrate-binding modules (CBMs) connected by linker sequences^1^,^2^. In the most recent update of the Carbohydrate-Active enZYmes database, CBMs were classified into 71 families based on amino acid sequence similarity (http://www.cazy.org)^3^. Some CBM families are classified into subfamilies based on key residues in the ligand binding site (e.g. CBM3^4^) or topological structure of the ligand binding region (e.g. CBM2^5^). However, an alternative classification based on the structure of the ligand binding site grouped these protein modules into three types: surface-binding, glycan-chain-binding, and small sugar-binding (Types A–C), respectively^1^. Recently, Gilbert *et al*. (2013) expanded on this classification and proposed that Type A enzymes recognize the surface of crystalline polysaccharides, Type B bind the internal regions of glycan chains (endo-type), and Type C bind the termini of glycans (exo-type)^6^.

The main function of CBMs is to recognize and bind polysaccharides and to enhance the hydrolytic activity of the appended hydrolase against insoluble substrates by increasing the effective enzyme concentration at the substrate surface^1^,^6^,^7^. Aromatic amino acids within the ligand binding site of CBMs play an important role in ligand recognition through hydrophobic interactions^1^,^8^. Some CBMs disrupt the ordered structure of recalcitrant substrates and increase their accessibility to the appended enzymes^9^,^10^,^11^,^12^.

Novel CBM families are frequently discovered and added to the appropriate databases. Recently, CBM66 was found to confer exolevanase activity on non-specific fructosidase through an avidity mechanism in which the CBM and CM target the termini of different branches of the same polysaccharide molecules^13^. The binding of expansin EXLX1, a representative member of CBM63, to whole cell walls is mediated by electrostatic and polar interactions between the basic D2 domain and the acidic polysaccharide matrix^14^. However, the crystal structure of the EXLX1-cellohexaose complex revealed that one cellohexaose molecule is packed between the aromatic surfaces of the D2 domains of two neighbouring EXLX1 molecules in a unique protein:ligand arrangement^15^. Identification of other novel family of CBMs may reveal further novel CBM-ligand interaction mechanisms.

Metagenomic approaches have been widely applied to the discovery of novel biocatalysts from diverse environmental samples^16^. CBM59, which binds efficiently to mannan, xylan and cellulose, was identified from metagenomes of cow manure samples^17^, and a novel starch-binding domain belonging to CBM69 was identified from the α-amylase derived from a marine metagenomic library^18^.

In our previous work, we identified a novel CBM from the C-terminus of endoglucanase C5614-1 (GenBank: ACA61140) derived from an uncultured microorganism present in buffalo rumen. CBM_C5614-1_ is composed of 189 amino acids, shares no significant homology to known CBMs, and can bind to a broad range of soluble polysaccharides such as barley glucan, methylcellulose, 2-hydroxyethycellulose, birch wood xylan, and carboxymethyl cellulose (CMC), as well as insoluble polysaccharides such as Avicel, acid swollen cellulose (ASC) and lichenan^19^.

In the present study, CBM_C5614-1_ and its homologs were found to constitute a novel family of CBMs (CBM72) with a broad ligand-binding specificity. Three key conserved aromatic residues in the ligand binding site were identified and characterized.

## Results

### Residues 364-497 of CBM_C5614-1_ constitute the polysaccharide binding region

In our previous work, we identified a novel CBM (189 aa) at the C-terminus (residues 349–537) of endoglucanase C5614-1 derived from an uncultured microorganism present in buffalo rumen^19^. Analysis of the isoelectric point (pI) of CBM_C5614-1_ with DNAStar revealed that the 40 aa basic C-terminal domain (BTD) has a pI of 9.58, while the pI of the remaining 149 aa is 4.5. In order to determine the shortest functional region capable of ligand binding, N- and C-terminal truncations of CBM_C5614-1_ were constructed. Binding experiments of deletion mutants showed that deletion of the C-terminal BTD did not affect the binding capacity towards soluble or insoluble polysaccharides, and neither did removing 15 aa from the N-terminus. However, removing 30 aa from the N-terminus resulted in a complete loss of binding to soluble polysaccharides. Residues 364–497 were therefore confirmed to house the ligand-binding region of CBM_C5614-1_ (Supplementary Fig. S1). Unless otherwise stated, all subsequent mention of CBM_C5614-1_ below refers to residues 364–497 of endoglucanase C5614-1.

### Sequence analysis of CBM_C5614-1_ homologs

A BLAST search using residues 364–497 identified seven homologous peptides in the NCBI protein database sharing 28–99% identity and 48–99% similarity with CBM_C5614-1_. All homologous peptides were found to be located at the C-terminus of glycoside hydrolases from uncultured microorganisms present in the rumen^20^,^21^,^22^,^23^. Of the seven homologs, AEK98797 (GeneBank number) is the most closely related, with only a single amino acid difference. ADR64668 and AFN57700 contained two tandem homologous regions, which were named ADR64668-1(549-680), ADR64668-2(411–546), AFN57700-1(520–651) and AFN57700-2(384–518), respectively. Unlike the other homologs, ADR64664 has an incomplete C-terminus.

**Figure 1:**
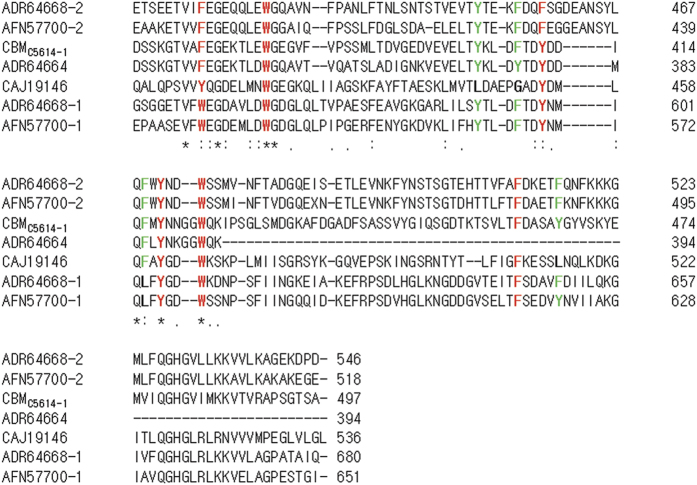
Multiple sequence alignment of CBM_C5614-1_ and selected homologs. Sequence identity and similarity are indicated by asterisks and dots, respectively. Completely conserved aromatic amino acid residues are red, and partially conserved aromatic amino acid residues are green. Numbers behind the each alignment represent the position of amino acids in the source enzyme of each member of the novel carbohydrate binding module family.

Aromatic amino acids within the ligand binding site are known to play an important role in recognizing and binding polysaccharides in all CBMs^1^. Sequence alignment revealed six aromatic amino acids that are completely conserved in all homologs, including Phe-372, Trp-380, Tyr-411, Tyr-418, Trp-423 and Phe-462 (numbered according to source enzyme C5614-1 of CBM_C5614-1_) (Fig. 1, red). Four partially conserved aromatic amino acids were also identified (Tyr-404, Phe-408, Phe-416 and Tyr-467) (Fig. 1, green).

### Representative CBM_C5614-1_ homologs also exhibited broad ligand specificity

**Figure 2:**
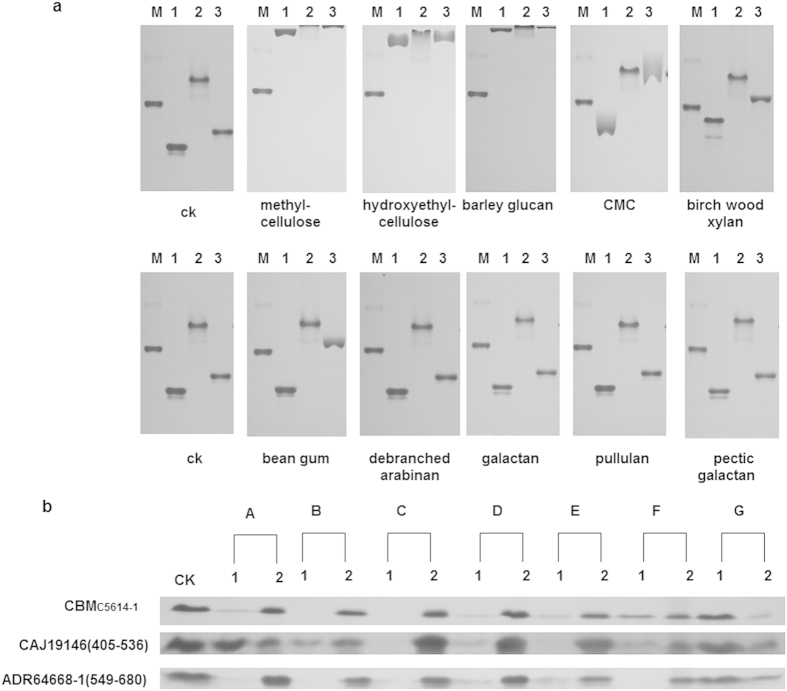
Binding of CBM_C5614-1_ and its homologs to polysaccharides. (**a**) Binding of CBM_C5614-1_ and its homologs to soluble polysaccharides. Proteins and BSA were separated using non-denaturing polyacrylamide gels containing 0.1% (wt/vol) soluble polysaccharides. A gel without polysaccharides (CK) served as a control. M: BSA control (no polysaccharides bound); 1: CBM_C5614-1_; 2: CAJ19146 (405–536); 3: ADR64668-1 (549–680). (**b**) Binding of CBM_C5614-1_ and its homologs to insoluble polysaccharides. 30 μg of purified CBM_C5614-1_ and its homologs were incubated with 200 μl 4% (wt/vol) insoluble polysaccharide including Avicel (**A**), ASC (**B**), insoluble birch wood xylan (**C**), mannan **(D**), lichenan (**E**), raw starch from cassava (**F**) or agarose (**G**). The same amount of protein used in the binding assay but without polysaccharide was included as a control (CK). After centrifugation, unbound protein in the supernatant (lane 1) and bound proteins in the precipitate (lane 2) were analyzed by SDS-PAGE.

ADR64668-1(549–680) and CAJ19146(405–536) that share 35% and 28% sequence identity with CBM_C5614-1_, respectively, were expressed in *Escherichia coli (E. coli)*, and the ligand binding capacity of the recombinant proteins was compared with that of CBM_C5614-1_. The homologs exhibited the same broad polysaccharide binding profiles (Fig. 2). Affinity gel electrophoresis revealed extensive binding to methyl cellulose, hydroxyethyl cellulose and barley glucan, weak binding to birch wood xylan, CMC and bean gum, and no binding to de-branched arabinan, galactan, pullulan and pectic galactan. Only CBM_ADR64668-1_ showed significant binding to CMC and bean gum (Fig. 2a). CBM_C5614-1_ and both homologs could bind to the insoluble polysaccharides Avicel, ASC, mannan, lichenan, birch wood xylan, and raw starch from cassava, but not to agarose (Fig. 2b). These results confirmed that the homologs were indeed CBMs.

### Three conserved aromatic amino acids play an important role in ligand binding in CBM72 proteins

**Figure 3:**
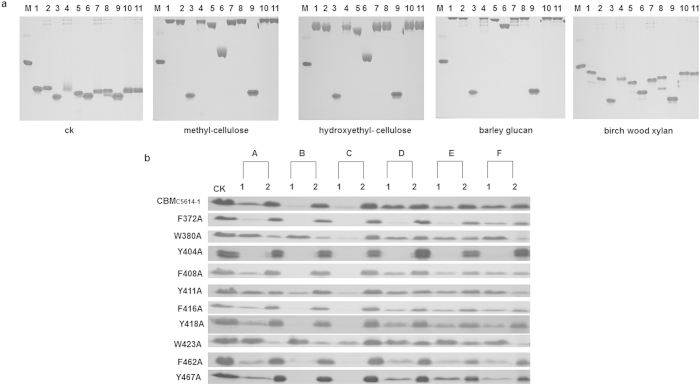
Binding of CBM_C5614-1_ variants to polysaccharides. (**a**) Binding of CBM_C5614-1_ variants to soluble polysaccharides. Proteins and BSA were separated using non-denaturing polyacrylamide gels containing 0.1% (wt/vol) soluble polysaccharides. A gel without polysaccharides served as a control (CK). M: BSA non-binding control; 1: CBM_C5614-1_; 2: F372A; 3: W380A; 4: Y404A; 5: F408A; 6: Y411A; 7: F416A; 8: Y418A; 9: W423A; 10: F462A; 11: Y467A. (**b**) Binding of CBM_C5614-1_ variants to insoluble polysaccharides. 30 μg of purified protein was incubated with 200 μl 4% (wt/vol) insoluble polysaccharide including Avicel (**A**), ASC (**B**), insoluble birch wood xylan (**C**), mannan (**D**), lichenan (**E**) and raw starch from cassava (**F**). The same amount of protein used in the binding assay but without polysaccharide was included as a control (CK). After centrifugation, unbound protein in the supernatant (lane 1) and bound proteins in the precipitate (lane 2) were analyzed by SDS-PAGE.

The 10 conserved aromatic amino acids of CBM_C5614-1_ were substituted with alanine using an overlap PCR method, mutant constructs were expressed in *E. coli*, and recombinant proteins were tested for binding to polysaccharides. Affinity gel electrophoresis revealed that CBM_C5614-1_ mutants W380A and W423A completely lost their ability to bind soluble polysaccharides, the affinity of Y411A was also diminished, however the other seven mutants displayed only a slight decrease in affinity or none at all, compared with wild type CBM_C5614-1_ (Fig. 3a).

The binding capacity of W380A and W423A to insoluble polysaccharides decreased dramatically and that of Y411A also decreased to some degree (Fig. 3b). In contrast, the other seven mutants had no obvious change compared with wild type CBM_C5614-1_. Trp-380, Tyr-411 and Trp-423 are therefore important for polysaccharide binding.

Studies by Sunna *et al*.^24^ and Hachem *et al*.^25^ suggested that 25% sequence identity is an appropriate threshold for CBM family members. CBM_C5614-1_ and its homologs all share more than 25% sequence identity with each other. The three key aromatic amino acids involved in substrate binding, are well conserved among all the homologs. These proteins therefore form a novel family of CBMs that we propose to call the CBM72 family. The broad substrate binding specificity suggests that CBM72 members belong to type B (endo-type) CBMs that bind internally to glycan chains^6^.

### Quantitative binding of CBM_C5614-1_ and its mutants to insoluble cellulose

**Figure 4:**
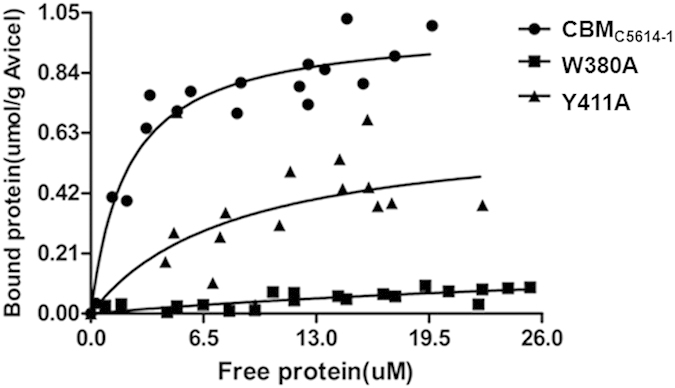
Depletion isotherms of wild type CBM_C5614-1_ and its mutants binding to Avicel. Binding isotherms were carried out as described in Materials and Methods.

The dissociation constant (*K*_*d*_) of wild type CBM_C5614-1_ binding to Avicel was 2.1, which was comparable to *K*_*d*_ values of other CBMs including CBM10, CBM3, CBM63, CBM17 and CBM28^15^. Affinity for Avicel was too low to determine the *K*_*d*_ of mutant W423A, but W380A exhibited a 20-fold decrease in affinity, and Y411A displayed a 4-fold reduction, compared to wild type CBM_C5614-1_ (Fig. 4; Table 1).

### Secondary structure determination of CBM_C5614-1_ and its mutants by CD spectroscopy

The secondary structure of CBM_C5614-1_ and its mutants was determined by CD spectroscopy. The composition of the secondary structures of mutants W380A, Y411A and W423A was only slightly different to that of wild type CBM_C5614-1_ (Supplementary Fig. S2; Supplementary Table S2), indicating that these conserved aromatic residues do not contribute to maintaining the protein structure and may be the key amino acids in the ligand binding site. CBM_C5614-1_ contains 44% β-strands structure. This high proportion of β-strands is consistent with other known CBMs^1^.

## Discussion

This study describes a novel CBM family, CBM72, members of which that display a particularly broad ligand binding specificity and target a range of β-linked soluble and insoluble polysaccharides that adopt very different conformations, including cellulose, β-1,3/1,4-mixed linked glucans, xylan, and mannan. Generally, CBMs that bind β-glucan chains often display broad specificity and recognize β-1,4-glucans (cellulose), β-1,3/1,4-mixed linked glucans, xyloglucan and other β-1,4-glycans, examples of which include CBM family 6^26^, 60^27^, 62^28^, and 65^29^,^30^. CBM72 proteins can bind to mannans (O3-C3-C2-O2 torsion angle of mannose = -60°) and β-1,4-glucans and -xylan (O3-C3-C2-O2 torsion angle of monosacccharide = + 60°)^27^, whereas other CBMs such as CBM60 can target the key geometric signature of β-1,4-glucans and β-1,4 xylan, but are not capable of binding to mannan^27^. This indicates that the binding site of CBM72 members may possess greater plasticity in order to accommodate very different ligand conformations.

The representative source enzyme endoglucanase C5614-1 of CBM72 showed the same broad substrate specificity observed previously^19^. C5614-1 can hydrolyze different β-1,4-linked polysaccharides but is most active towards barley glucan, followed by CMC, lichenan, 2-hydroxyethyl cellulose, methyl cellulose and xylan^19^. Generally, CBMs do not increase the hydrolytic activity of appended catalytic modules towards soluble substrates^31^,^32^. In some cases, the CBM truncated mutant Egl330 showed improved turnover rate (*k*_cat_) and catalytic efficiency (*k*_cat_/*K*_*m*_) with CMC as substrate compared with wild type Egl499^33^. In our previous study, deletion of CBM_C5614-1_ from endoglucanase C5614-1 increased its hydrolytic activity against barley glucan^19^, despite the high affinity of CBM_C5614-1_ for this substrate, suggesting that this CBM hindered its catalytic module to degradation of barley glucan. This result indicates that there may be no correlation between hydrolytic efficiency of the appended catalytic module and binding capacity of the CBM towards soluble substrates.

We next investigated how CBM72 is able to bind a broad range of ligands, and considered the possibility of more than one binding sites on the surface for this purpose. CBMs in family 6 were reported to include three ligand binding sites with distinct specificities, and these proteins also bind a broad ligand range including β1,4-1,3-mixed linked glucans, cello-oligosaccharides, insoluble forms of cellulose, the β1,3-glucan laminarin, and xylooligosaccharides^26^,^34^,^35^. Mutagenesis of the conserved aromatic amino acids in CBM_C5614-1_ showed that Trp380 and Trp423 were crucial for binding barley glucan, 2-hydroxyethyl cellulose, birch wood xylan and Avicel, suggesting that all these diverse ligands were bound to the same binding site of CBM_C5614-1_. The basis for the broad binding specificity remains unknown without structural data, and determining the structure of a CBM72-ligand complex is a priority in future studies.

The biological function of the unusually broad ligand specificity of CBM72 proteins also remains unknown, but targeting and proximity effects of CBMs may play an important role. Herve *et al*. (2010) demonstrated that CBMs can potentiate the action of appended catalytic modules toward polysaccharides in intact cell walls through the recognition of non-substrate polysaccharides, provided that the non-substrate and substrate are close to each other. For example, the capacity of xylanases to degrade xylan in secondary walls was potentiated by both xylan and cellulose-directed CBMs^36^. The plant cell wall is highly complex and is comprised of various polysaccharides such as cellulose, pectin and hemicellulose that crosslink with each other to form an intricate meshwork^37^. Catalytic modules attached to CBMs with broad ligand specificity may be easier to target to their intended substrates than those appended to CBMs with strict ligand specificity.

In conclusion, this study reports a new family of CBMs with a uniquely broad ligand binding specificity. Three conserved CBM aromatic amino acids were found to play an important role in recognizing and targeting ligands. The presence of CBM72 domains may facilitate the targeting of catalytic domains to their substrates in plant cell walls. These novel CBMs can provide new insight into CBM-ligand interactions and may have potential for engineering of cellulase or other enzymes for improved biotechnological performance.

## Materials and Methods

### Sequence analysis

Protein modular structure was predicted using SMART (http://smart.embl-heidelberg.de) and Pfam (http://pfam.xfam.org/search/sequence). Multiple alignments of CBM_C5614-1_ and its homologs were performed using ClustalW (http://www.ebi.ac.uk/Tools/ClustalW).

### Sources of carbohydrates

Carboxymethyl cellulose, birch wood xylan, hydroxyethyl cellulose, methyl cellulose, bean gum and Avicel were purchased from Sigma (St. Louis, MO). Cello-oligosaccharides, barley glucan, galactan, debranched arabinan, pullulan, pecticgalactan and lichenin were purchased from Megazyme international Ireland Ltd (Bray, Ireland).

### Cloning and expression of wild type and mutant CBM_C5614-1_ and its homologs

The cosmid C5614-1 (GenBank: EU449484), derived from metagenomics experiments on ruminal microbiomes^38^, were used as templates for amplifying the gene encoding CBM_C5614-1_. DNA fragments were amplified using Primestar polymerase (TaKaRa, Kyoto, Japan) and an appropriate combination of primers (Supplementary Table S1) that included NdeI or XhoI restriction sites (underlined), and amplified products were purified, digested with NdeI and XhoI, and cloned into the expression vector pET-30a( + ) (Novagen, Darmstadt, Germany) in order to express recombinant proteins with 6 × His tags at the C-terminus.

Genes encoding CBM_ADR64668-1_ (549–680) and CBM_CAJ19146_ (405–536) were synthesized artificially based on the DNA sequences of CBM_C5614-1_ homologs (GenBank: ADR64668, CAJ19146) following codon optimization to maximize expression in *E. coli*, and ligated into the NdeI and XhoI restriction sites of expression vector pET-30a(+).

Constructs encoding deletion derivatives of CBM_C5614-1_ (349–537) were constructed by deleting the nucleotides encoding successively the N-terminal 15 aa, and the C-terminal 40 aa, using PCR. The following constructs were prepared: CBM_C5614-1_ (349–497), CBM_C5614-1_ (364–497) and CBM_C5614-1_ (379–497), where numbers in brackets correspond to the amino acid sequence of endoglucanase C5614-1. PCR products were ligated into the expression vector pET-30a( + ). Primers used for PCR amplification are shown in Supplementary Table S1.

Variants of CBM_C5614-1_ were engineered by site-directed mutagenesis using an overlap-extension PCR procedure based on that described by Ho *et al*.^39^ using primers shown in Supplementary Table S1.

### Purification of recombinant proteins

All constructs were verified by DNA sequencing and transformed into *E. coli* Rosetta (DE3) pLysS (Novagen) for protein expression and positive clones were selected by growth on kanamycin- and chloramphenicol-containing media. Cells harboring the recombinant plasmids were grown to an OD_600_ of 0.6 in LB broth containing 25 μg/mL kanamycin and 34 μg/mL chloramphenicol at 37 °C with shaking at 200 rpm. The expression of the target genes was induced by adding 0.5 mM isopropyl-β-D-1-thiogalactopyranoside to the medium and continuing the incubation at 20 °C with shaking at 100 rpm overnight. Recombinant proteins were extracted from the cytoplasmic fraction of cell lysates and purified by affinity chromatography with Nickel-nitrilotriacetic acid agarose resin (Ni-NTA, Qiagen) according to the manufacturer’s instructions. Purified proteins were desalted using Amicon Ultra-10 ultrafiltration columns (Millipore, Billerica, MA) and diluted into citrate/phosphate buffer, pH 5 (a mixture of 100 mM citric acid and 200 mM Na_2_HPO_4_ at a volume ratio of 97:103).

### Protein determination

Protein concentration was determined using the Micro BCA kit (Pierce, Rockford, IL) with bovine serum albumin (BSA) as the standard. Sodium dodecyl sulfate-polyacrylamide gel electrophoresis (SDS-PAGE) was performed on 12% polyacrylamide gels using the method of Laemmli^40^ and proteins were visualized by Coomassie Blue staining.

### Soluble polysaccharide binding assays

The capacity of CBM_C5614-1_, its homologs and variants to bind to soluble polysaccharides was determined by affinity gel electrophoresis performed as described by Duan *et al*.^19^. Polysaccharides were incorporated into the gel at a concentration of 0.1% prior to polymerization. A control gel without polysaccharides was prepared and run simultaneously. Electrophoresis was conducted at 100 V for 4 h at 4 °C. BSA that does not bind polysaccharides was used as a negative control.

### Insoluble polysaccharide binding assays

Proteins (30 μg) were mixed with 4% (wt/vol) insoluble polysaccharides in 0.2 mL citrate/phosphate buffer (pH 5) and incubated on ice for 5 h with occasional stirring. After centrifugation at 10,000 g, 4 °C for 10 mins, supernatants (unbound proteins) were collected and pellets were washed twice with 1 mL citrate/phosphate buffer (pH 5). Polysaccharides bound to proteins were then eluted with 100 μL 2% SDS for 30 min at 37 °C. Eluted proteins were collected by centrifugation and subjected to SDS-PAGE. Controls with proteins but no ligands were included to ensure that precipitation did not occur during the assay.

Depletion isotherms to quantify the binding of wild type CBM_C5614-1_ and its variants to Avicel were carried out by mixing protein 1–100 μM protein with 0.2 mL citrate/phosphate buffer (pH 5) containing 1% Avicel. The mixture was shaken on a table concentrator (TENSUG) at 340 rpm, 4 °C until equilibrium was reached (5 h). Samples were centrifuged at 10,000 g, 4 °C for 2 min to pellet the bound substrate, and unbound proteins in the supernatant were quantified using the Pierce BCA protein assay kit using the formula: bound protein = total protein—unbound protein. Dissociation constants (*K*_d_) and Bmax values (amount of protein bound at saturation) were calculated by fitting the data to a single site Langmuir isotherm using Graphpad Prism 5 (GraphPad Software, Inc., San Diego, CA). At least three separate binding isotherms were carried out for each protein.

### Circular Dichroism (CD) Spectroscopy

Proteins were dialyzed extensively against 5 mM sodium phosphate (pH 5) and CD spectra were collected on a Biologic MOS-450 spectropolarimeter between 188–250 nm with steps of 60 nm per minute. All samples were analyzed in triplicate. After subtraction of buffer spectra, CD spectra were smoothed using the means-movement method and analyzed using CDROM Biokine 4 and Origin 8.0 that utilize the K2D method^41^ to analyze protein secondary structure.

## Additional Information

**How to cite this article**: Duan, C.-J. *et al*. Identification of a novel family of carbohydrate-binding modules with broad ligand specificity. *Sci. Rep.* **6**, 19392; doi: 10.1038/srep19392 (2016).

## Supplementary Material

Supplementary Information

## Figures and Tables

**Table 1:** Affinity of wild type and variants of CBM_C5614-1_ for Avicel as determined by depletion isotherms.

Protein	K_d_	B_max_
	μM	μmol/g Avicel
CBMC_C5614-1_	2.10	1.00
W380A	43.67	0.23
Y411A	8.22	0.65
W423A	a	a

^a^Too low to be determined accurately.
