# Supplementary material for: Identification of a novel family of carbohydrate-binding modules with broad ligand specificity
Source: Sci Rep. 2016 Jan 14;6:19392. doi: 10.1038/srep19392 (PMC4725902; doi:10.1038/srep19392)
Supplement: Supplementary Information [file srep19392-s1.pdf]

Supplementary Information

Identification of a novel family of carbohydrate-binding modules with broad ligand specificity

Cheng-Jie Duan*, Yu-Liang Feng, Qi-Long Cao, Ming-Yue Huang, Jia-Xun Feng*

Supplementary files provided with this submission:

Figure S1. Construction and binding capacity of three $CBM_{C5614-1}$ deletion mutants.

Figure S2. The circular dichroism spectra of $CBM_{C5614-1}$ and its variants.

Table S1. Primer pairs used in this work.

Table S2. The secondary structure about $CBM_{C5614-1}$ and its variants, the data was analyzed using K2D by Dicroprot.

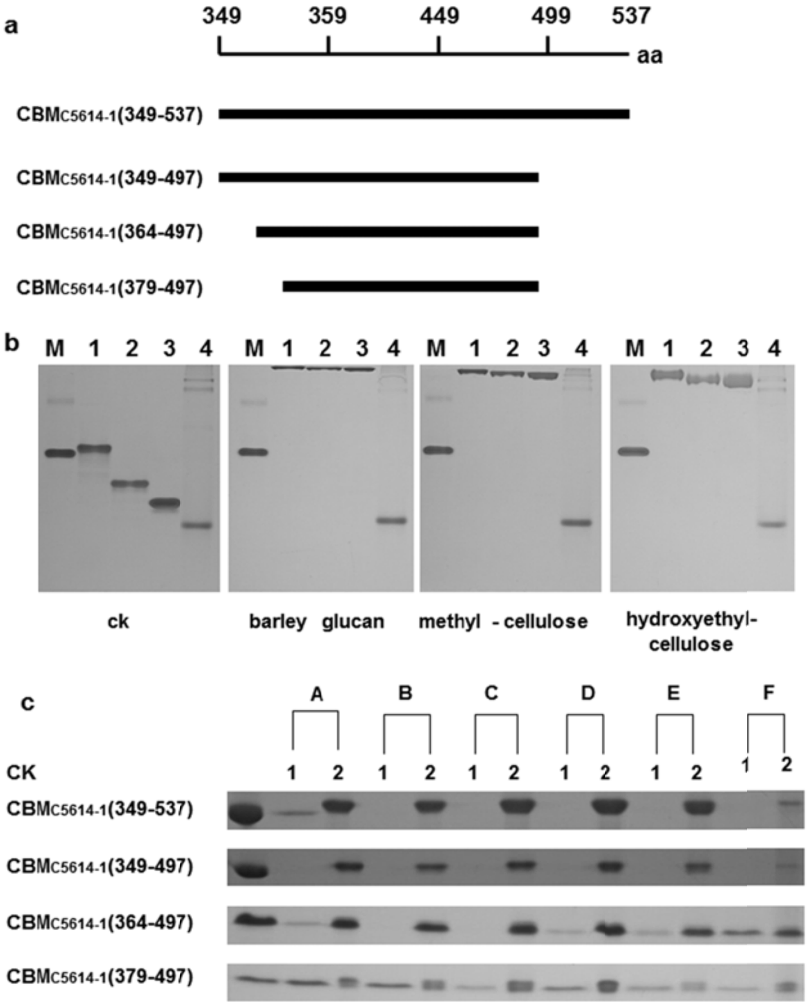

Figure S1. Construction and binding capacity of three CBM$_{C5614-1}$ deletion mutants.
a: Construction of CBM$_{C5614-1}$ deletion mutants. Numbers in brackets refer to the amino acid sequence of the source enzyme C5614-1 of CBM$_{C5614-1}$.
b: Binding of CBM$_{C5614-1}$ deletion mutants to soluble polysaccharides. Proteins and BSA were separated using non-denaturing polyacrylamide gels containing 0.1% (wt/vol) soluble polysaccharides. A gel without polysaccharides served as a control (CK). Lane M, BSA (negative control); Lane 1, wild type CBM$_{C5614-1}$ (349-537) (positive control). Lane 2, CBM$_{C5614-1}$ (349-497); Lane 3, CBM$_{C5614-1}$ (364-497); Lane 4, CBM$_{C5614-1}$ (379-497).
c: Binding of CBM$_{C5614-1}$ deletion mutants to insoluble polysaccharides. Deletion mutants were incubated for 4 h with insoluble polysaccharides in the form of Avicel (A), ASC (B), insoluble birch wood xylan (C), mannan (D), lichenan (E) and raw starch from cassava (F). The same amount of protein used in the binding assay but without polysaccharide was included as a control (CK). After centrifugation, unbound protein in the supernatant (lane 1) and bound proteins in the precipitate (lane 2) were analyzed by SDS-PAGE.

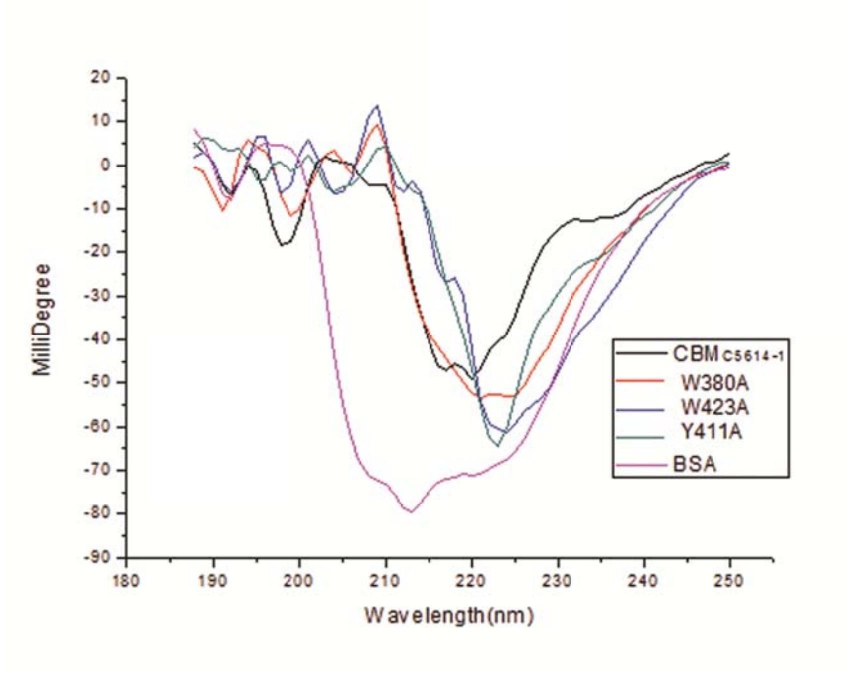

Figure S2. The circular dichroism spectra of CBM$_{C5614\text{-}1}$ and its variants. The circular dichroism spectra were recorded as described under “Materials and Methods.”

Table S1 Primer pairs used in this work.

Primer pairs	Sequence (5' to 3')	Product/product length
$CBM_{C5614-1}$F1/ $CBM_{C5614-1}$R1	GGGCATATGAAAGCCTATCATGGCAGCGCGTTC/ GACCTCGAGTTGTGCTATGTATTTTTGCCGTTCTGG	*$CBM_{C5614-1(349-537)}$*/564 bp
$CBM_{C5614-1}$F1/ $CBM_{C5614-1}$1-149 R1	As above/GACCTCGAGTGCTGACGTACCGGAAGGTGCTCTTA	*$CBM_{C5614-1(349-497)}$*/447 bp
$CBM_{C5614-1}$16-149 F1/ $CBM_{C5614-1}$1-149 R1	AGCCATATGGATTCTTCGAAGGGTACTGTTGCTTTTG /as above	*$CBM_{C5614-1(364-497)}$*/402bp
$CBM_{C5614-1}$31-149F1/ $CBM_{C5614-1}$1-149 R1	AGCCATATGGAATGGGGAGAAGGCGTGTTCGTTC/As above	*$CBM_{C5614-1(379-497)}$*/357bp
$CBM_{C5614-1}$F372AF1/ $CBM_{C5614-1}$1-149R1	AGGCATATGGATTCTTCGAAGGGTACTGTTGCT**GCA**GAAGGCGAGAAGAC/as above	Mutant *F372A*/402 bp
$CBM_{C5614-1}$16-149F1/ $CBM_{C5614-1}$W380FR1	As above/GAACACGCCTTCTCC**TGC**TTCCAACGTCTTCTCGCCTT	*Forward fragment of* mutant*W380A*/66 bp
$CBM_{C5614-1}$W380AF1/ $CBM_{C5614-1}$1-149 R1	AAGGCGAGAAGACGTTGGAA**GCA**GGAGAAGGCGTGTTC/As above	*Backward fragment of* mutant*W380A*/374 bp
$CBM_{C5614-1}$16-149F1/ $CBM_{C5614-1}$Y404AR1	As above/TCGGTGAAGTCGAGTTT**TGC**GGTCAGTTCTACTTCCAC	*Forward fragment of* mutant*Y404A*/140 bp
$CBM_{C5614-1}$Y404AF1/ $CBM_{C5614-1}$1-149 R1	GTGGAAGTAGAACTGACC**GCA**AAACTCGACTTCACCGA /As above	*Backward fragment of* mutant*Y41A* /300 bp
$CBM_{C5614-1}$16-149F1/C $BM_{C5614-1}$F408AR1	As above/TATCATCATAGTCGGT**TGC**GTCGAGTTTATAGGTCAGTTC	*Forward fragment of* mutant*F408A* / 151 bp
$CBM_{C5614-1}$F408AF1/ $CBM_{C5614-1}$1-149 R1	GAACTGACCTATAAACTCGAC**GCA**ACCGACTATGATGATA /As above	*Backward fragment of* mutant*F408A* / 291 bp
$CBM_{C5614-1}$16-149F1/ $CBM_{C5614-1}$Y411FR1	As above/CATGAACTGAATATCATC**TGC**GTCGGTGAAGTCGAGTTTATAGG	Forward fragment of mutant *Y411A*/ 162 bp
$CBM_{C5614-1}$Y411AF1 / $CBM_{C5614-1}$1-149 R1	CCTATAAACTCGACTTCACCGAC**GCA**GATGATATTCAGTTCATG /As above	Backward fragment of mutant *Y411A*/284 bp
$CBM_{C5614-1}$16-149F1/ $CBM_{C5614-1}$F416AR1	As above/CCATTATTATACAT**AGC**CTGAATATCATCATAGTCGGTGAAGTCGAG	Forward fragment of mutant *F416A*/ 173 bp
$CBM_{C5614-1}$F416AF1/C $BM_{C5614-1}$1-149 R1	CTCGACTTCACCGACTATGATGATATTCAG**GCT**ATGTATAATAATGG /As above	Backward fragment of mutant *F416A*/278 bp
$CBM_{C5614-1}$16-149F1/ $CBM_{C5614-1}$Y418AR1	As above/TCCACCATTATTATACAT**AGC**CTGAATATCATCATAGTCGGTGAA	Forward fragment of mutant *Y418A*/ 183 bp
$CBM_{C5614-1}$Y418AF1/ $CBM_{C5614-1}$1-149 R1	TTCACCGACTATGATGATATTCAG**GCT**ATGTATAATAATGGTGGA /As above	Backward fragment of mutant *Y418A*/270 bp
$CBM_{C5614-1}$16-149F1/C $BM_{C5614-1}$W423AR1	As above/GAAAGACCACTGGGTATCTTCTG**TGC**TCCACCATTATTATAC	Forward fragment of mutant *W423A*/ 203 bp
$CBM_{C5614-1}$W423AF1/ $CBM_{C5614-1}$1-149 R1	TAATGGTGGA**GCA**CAGAAGATACCCAGTGGTCTTTC /As above	Backward fragment of mutant *W423A*/ 235 bp

$CBM_{C5614-1}$16-149F1/ $CBM_{C5614-1}$F462AR1	As above/CCATAAGCCGAGGCATC**TGC**AGTCAAGACCGAT	Forward fragment of mutant *F462A*/ 314 bp
$CBM_{C5614-1}$F462AF1/ $CBM_{C5614-1}$1-149 R1	ATCGGTCTTGACT**GCA**GATGCCTCGGCTTATGG/As above	Backward fragment of mutant *F462A*/ 121 bp
$CBM_{C5614-1}$16-149F1/ $CBM_{C5614-1}$Y467AR1	As above/AGAAACATATCC**TGC**AGCCGAGGCATCGAAGTC	Forward fragment of mutant *Y467A*/324 bp
$CBM_{C5614-1}$Y467AF1/ $CBM_{C5614-1}$1-149 R1	GACTTTCGATGCCTCGGCT**GCA**GGATATGTTTCT/As above	Backward fragment of mutant *Y467A*/112 bp

Added restriction sites are underlined.
The mutant codons are shown in bold.

Table S2. The secondary structure about $CBM_{C5614\text{-}1}$ and its variants, the data was analyzed using K2D by Dicroprot.

Folding type	BSA	$CBM_{C5614\text{-}1}$	W380A	Y411A	W423A
ALPHA	≥0.30	≥0.08	≥0.07	≥0.06	≥0.05
BETA	≥0.13	≥0.44	≥0.46	≥0.47	≥0.47
RANDOM	≥0.56	≥0.48	≥0.48	≥0.48	≥0.48
